# Stereolithographic Surgical Guide with a Combination of Tooth and Bone Support: Accuracy of Guided Implant Surgery in Distal Extension Situation

**DOI:** 10.3390/jcm9030709

**Published:** 2020-03-05

**Authors:** Chia-Cheng Lin, Masahiro Ishikawa, Takeo Maida, Hsin-Chung Cheng, Keng-Liang Ou, Takashi Nezu, Kazuhiko Endo

**Affiliations:** 1 Division of Biomaterials and Bioengineering, School of Dentistry, Health Sciences University of Hokkaido, Hokkaido 061-0293, Japan; 2 School of Dentistry, College of Oral Medicine, Taipei Medical University, Taipei 110, Taiwan; 3 Department of Dentistry, Shin Kong Wu Ho-Su Memorial Hospital, Taipei 111, Taiwan; 4 Division of Oral and Maxillofacial Surgery, Department of Human Biology and Pathophysiology, Health Sciences University of Hokkaido, Hokkaido 002-8072, Japan; 5 Division of Advanced Prosthodontics, Department of Oral Rehabilitation, Health Sciences University of Hokkaido, Hokkaido 002-8072, Japan; 6 Department of Dentistry, Taipei Medical University-Shuang Ho Hospital, New Taipei City 235, Taiwan; 7 Department of Oral Hygiene Care, Ching Kuo Institute of Management and Health, Keelung 203, Taiwan; 8 Department of Dentistry, Taipei Medical University Hospital, Taipei 110, Taiwan; 9 3D Global Biotech Inc., New Taipei City 221, Taiwan

**Keywords:** guided surgery, stereolithographic surgical guide, computer-aided design/computer-aided manufacturing (CAD/CAM), distal free-end, accuracy, in-vitro research

## Abstract

A distal free-end situation could result in insufficient stability of the surgical guide, and could reduce accuracy of the static guided implant surgery (sGIS). The purpose of this study was to investigate the accuracy of sGIS using a combination tooth-and-bone supported stereolithographic (SLA) surgical guide in distal extension situation. Thirty dentists, each placed three implants at the Federal Dentaire Internationale (FDI) teeth positions #46, #47 (a distal extension situation), and #36 (a single tooth gap) via the surgical guide on a model fixed to a manikin. Pre- and post-operative computed tomography (CT) images of the models were superimposed, and the positional and angular deviations of the implants were measured with metrology software. An analysis of variance (ANOVA) test was performed to evaluate the intergroup differences. No significant differences were found for all the positional and angular deviations among the three implant sites, except the bucco-lingual deviation at the implant platform in the #47 position (0.43 ± 0.19 mm) that was significantly larger than the #46 (0.21 ± 0.14 mm) and #36 (0.24 ± 0.25 mm) positions (*p* < 0.0001). Within the limits of this study, we conclude that, in distal extension situation of missing mandibular molars, adding a bone-supported strut in the distal part of the surgical guide can be beneficial to the accuracy of the sGIS.

## 1. Introduction

The introduction of static guided implant surgery (sGIS) has been beneficial to optimize and facilitate the implant planning and positioning [1]. Using three-dimensional planning software, a virtual implant treatment plan can be made and transferred to the patient via surgical templates or surgical guides in implant surgery. The ideal implant position can be attained, and it helped to avoid damaging the surrounding anatomical structures [2]. Compared to freehand implant placement, the accuracy of sGIS has been shown to be superior in different clinical situations [2,3,4]. Kuhl et al. [5] reported that using printed templates for guided implant surgery could achieve a high accuracy in terms of implant positioning. However, although the mean values of implant deviations of sGIS were generally acceptable, relatively large maximal deviations were revealed in the literature [4,6]. Testori et al. [7] suggested that a safe distance of at least 2 mm was needed between implants and anatomic structures when planning computer-guided implant surgery.

Many variables could affect the accuracy of sGIS, from acquisition and the quality of the patient’s clinical and digital data, the digital planning software and procedure, fabrication of the surgical guide, to the execution of the guided surgery [1]. Among these variables, an important factor is the design of the surgical guide. Various designs have been developed according to the type of guide support, including tooth-supported, mucosa-supported, and bone-supported surgical guides [8]. The accuracy of sGIS using different designs of surgical guides has been investigated and compared. In a systematic review and meta-analysis study, Raico Gallardo et al. [9] concluded that the tooth-supported guides showed more accuracy compared with the bone-supported and mucosa-supported guides. Tahmaseb et al. [2] also reported a better accuracy of the tooth- and mucosa-supported guides compared to the bone-supported guides.

To achieve accuracy, a good fitting to the underlying tissue and the correct setting of the surgical guide during the surgery is crucial. The bone-supported surgical guides are commonly applied to patients who need more extensive bone surgery; therefore, the reflected flap may make the correct intraoperative positioning of the guide more difficult [9]. The tissue-supported surgical guides are mainly used for total edentulous patients, and the resilience and the thickness of the mucosa may affect the correct seating of the surgical guide [10,11]. Stabilized by the wide-ranging remaining teeth, the tooth-supported surgical guides are commonly applied to a tooth gap with limited missing teeth compared to the mucosa-supported and bone-supported surgical guides. This rigid support from the teeth provides a sound basis for the correct intraoral seating of the guide, which is of prime importance to attain accuracy.

Partial edentulism of the molars is one of the most common clinical situations requiring dental implant treatment. A tooth- or tooth-and-mucosa supported surgical guide with only one end supported on the remaining teeth is generally used for these cases. Although the tooth-supported guide was considered to be capable to achieve more accuracy, this distal free-end situation of the guide could result in insufficient stability, and thus reduced accuracy. Larger implant deviations by using such unilaterally supported surgical guides could be expected due to tilting and bending of the guide [12]. In an in vitro model experiment using sGIS, El Kholy et al. [13] reported that, compared to posterior implants placed in bilaterally tooth bounded sites, implants placed in distal extension situations led to significantly higher crestal and apical 3D deviations. Similar results have also been reported in other studies [14,15]. The risk to have more deviations will rise when the extension of the surgical guide increases, due to the bending effect of the surgical guide in the posterior region [6].

For these reasons, it is necessary to modify this unilaterally tooth-supported design to avoid the possible bending or tilting of the surgical guide during implant surgery. The authors advocated a design combined with tooth support and bone support, by adding a rigid bone-supported component at the free end of the unilaterally tooth-supported surgical guide. The stability of the guide could be increased due to the additional bone support at the distal extension end, thus the accuracy of the implant surgery could be potentially improved. The purpose of the present study was to investigate the accuracy of computer-guided implant surgery in distal extension situation, using this combination tooth-and-bone supported stereolithographic (SLA) surgical guide. The null hypothesis was that no significant difference would be found in the accuracy of sGIS between the bilaterally tooth-supported surgical guide and the combination tooth-and-bone supported surgical guide at the first molar site.

## 2. Materials and Methods

### 2.1. Surgical Guide Fabrication

**Figure 1:**
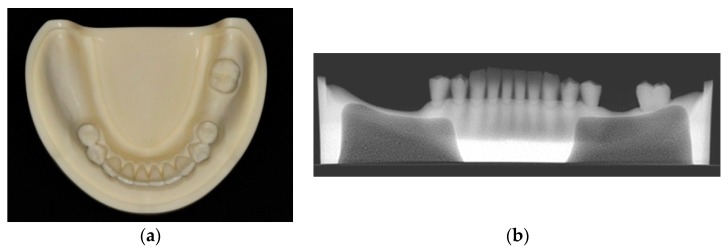
(**a**) The mandibular model used for the study. (**b**) The radiographic image of the model showed that the edentulous part had a two-layered construction to duplicate the bone anatomy.

Thirty urethane mandibular models (Nissin Dental Model P9-X.1523-L, Nissin Dental Products Inc., Kyoto, Japan) were used for this study. All the molars except the left second molar were missing, contributing to a distal extension situation in the right side and a single tooth gap in the left side. Artificial mucosa was not used for the edentulous ridge so as to eliminate the potential influence of the reflected flap. The edentulous part of the model had a two-layered construction to duplicate the bone anatomy with cortical bone structure outside and type II density cancellous bone inside (Figure 1).

**Figure 2:**
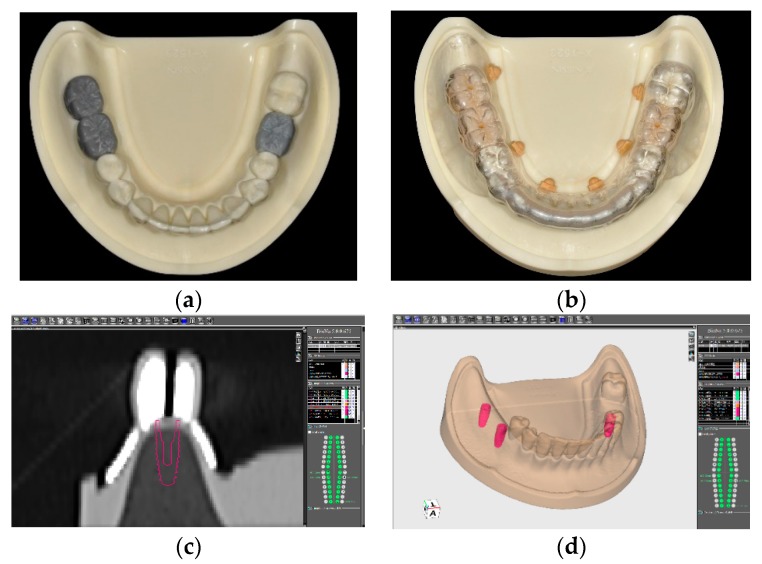
Preparation and planning of the surgical guide. (**a**) Wax-up of the missing teeth. (**b**) Radiographic scan template converted from the wax-up. (**c**) Based on the wax-up, an implant with proper length and diameter was selected. (**d**) Three implants were virtually planned at the missing molar positions.

One master model was used to make the surgical guide. A wax-up of the missing teeth were created on the model then converted into a radiographic scan template. After confirmation of the correct fitting of the scan template on the model, a computed tomography (CT) scan of the model with the scan template was taken with a multi-slice helical CT scanner (Aquilion 16, Toshiba, Tokyo, Japan). The Digital Imaging and Communication in Medicine (DICOM) files of the scan were imported into the implant simulation software (BioNa^®^, Wada Precision Dental Laboratories Co. Ltd., Osaka, Japan). Three Kyocera POIEX 3.7 by 10 mm implants (Kyocera Medical Co. Osaka, Japan) were virtually planned based on the wax-up at the missing molar positions, corresponding to the Federal Dentaire Internationale (FDI) teeth #47, #46, and #36 (Figure 2), and a surgical guide was designed according to the planned implant position.

**Figure 3:**
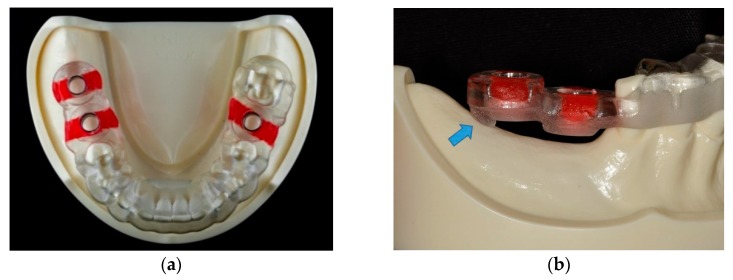
(**a**) The finished stereolithographic (SLA) surgical guide. (**b**) A supporting strut was designed in the distal part of the surgical guide to offer a combination tooth-and-bone support (blue arrow).

The surgical guide was bilaterally tooth-supported for the single tooth gap of tooth position #36 in the left side, and unilaterally tooth-supported for the distal extension situation of tooth position #46 and #47 in the right side. In the free-end right side, a supporting strut intimately contacting the edentulous ridge was designed in the distal part of the surgical guide to offer a combination tooth-and-bone support (Figure 3). Based on this virtual planning and design, six SLA surgical guides were manufactured using the modeling resin material (MED610 by Stratasys Ltd., Eden Prairie, MN, USA) and the same settings (3 mm thickness and 0.03 mm guide-to-teeth offset) with a 3D printer (Eden260VS Dental Advantage, Stratasys Ltd.) by a dental laboratory (Wada Precision Dental Laboratories Co. Ltd.).

### 2.2. Implant Placement

To ensure the surgical guides used for the study fit well to all the models, 3D surface scans were performed for each model with a desktop scanner (Swing, DOF Inc., Seoul, Korea). The images of the scans were compared with the image of the master model used to fabricate the surgical guide by a metrology software (Geomagic Control X, 3D Systems Inc., Rock Hill, SC, USA). Apart from the interdental spaces with undercuts, which could not be registered well, any model with a surface discrepancy larger than 0.05 mm was discarded until a total of 30 models were selected for implant placement.

Thirty dentists volunteered to participate in this study, including 15 experienced surgeons who had inserted more than 20 dental implants, and 15 dentists who never perform implant surgery in a real patient. Without screening in advance, all of the dentists happened to perform dental treatments with the right hand. The technical detail about the procedure of the guided-implant surgery were instructed to the dentists before the model surgery, and the surgical guide was checked for fit to the model through perceiving the inspection window. The model, along with the opposing maxillary model, was fixed to the manikin head to simulate clinical conditions. Because the manikin head was extending from a laboratory bench, the operators were positioned behind the manikin head during the simulation surgery. The SLA surgical guide, together with the guided surgical kit (Bone Navi System, Wada Precision Dental Laboratories Co. Ltd.), was used for the sequential drilling procedure according to the manufacturer’s recommendations.

**Figure 4:**
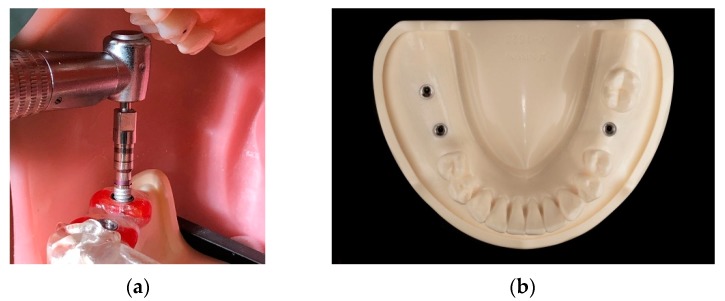
(**a**) Simulation implant placement performed on the model fixed to the manikin. (**b**) Three implants placed on the model.

Every surgical guide was used for five model surgeries only to decrease the influence of mechanical wear on the guide sleeves from repeated use according to the experiment results by Fernandez-Gil et al. [16]. The sleeves of the surgical guides were also visually examined to ensure there were no wear marks, after every simulation surgery. Moreover, for minimizing the errors from using dull drills during the osteotomy procedure, each dentist used a brand new drill for the final drilling procedure. After the osteotomy preparation, every dentist placed three implants (Kyocera POIEX 3.7 by 10 mm, Kyocera Medical Co.) with the handpiece, then manually with a ratchet wretch on one model via the SLA surgical guide. In total, 90 implants were placed (Figure 4).

### 2.3. Accuracy Analysis

After implant placement, all the models were checked with a cone beam computed tomography (CBCT) scanner (3D eXam, KaVo Dental GmbH, Biberach, Germany) to obtain the post-implantation images. The DICOM files of each post-implantation images were superimposed with the pre-implantation 3D virtual model using the registration tool of a software (BenQ AB Guided Service, Ashdod, Israel). These data were then exported in a Standard Tessellation Language (STL) format to a computer-aided design (CAD) software (Geomagic Design X, 3D Systems Inc.). To identify the direction of the implant deviation, the buccal direction of each implant site was designated perpendicular to the dental arch curve, and a corresponding coordinate system of each implant site was created according to the longitudinal axis and the designated buccal direction of the planned implant. 

The implant images of the post-implantation model were not well-defined at this point due to the effect of CBCT metal artifacts. Using the best-fit algorithm of the same software, these implant images were then replaced by STL files corresponding to the placed implant to more appropriately measure the implant deviation. These data were subsequently exported to the metrology software (Geomagic Control X, 3D Systems Inc.). The center of the implant platform, the implant apex, and the longitudinal axis of each planned and placed implant were determined with the software, and the deviations between the planned and placed implants could be correctly measured.

**Figure 5:**
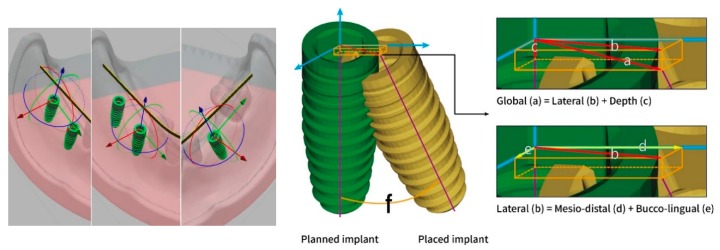
The coordinate systems of each implant site (left) were created according to the longitudinal axis and the designated buccal direction of the planned implants. Deviations of measurement at the implant platform were defined as: (a) global deviation, (b) lateral deviation, (c) depth deviation, (d) mesio-distal deviation, (e) bucco-lingual deviation, and (f) angular deviation. The deviations at the implant apex were defined and measured by the same coordinate system of each implant site.

**Figure 6:**
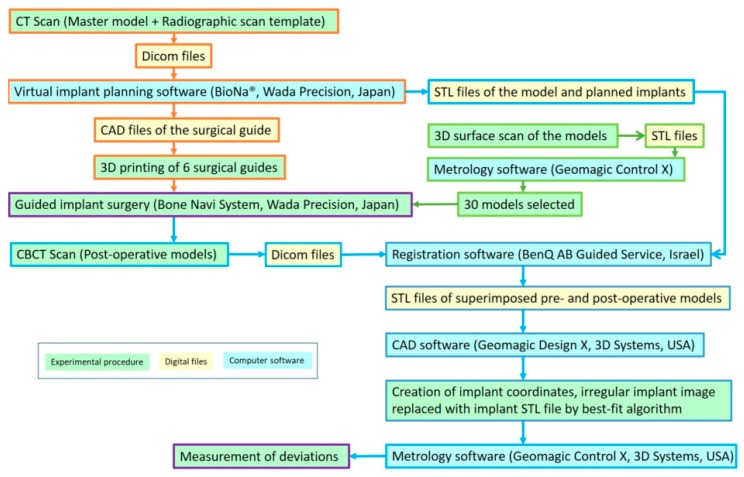
Summarized workflow of the experiment. Orange arrows: Procedure of surgical guide fabrication; green arrows: Procedure of model selection; and blue arrows: Procedure of deviation measurement.

The deviations between placed and planned implants were then measured according to the following definitions: The 3D distance of the center of the implant platform/apex between the planned and placed implants was defined as the global deviation at the implant platform/apex, and the 3D angle between the planned and placed implant axis was defined as the angular deviation. To further identify the direction of the deviations, the global deviation at the implant platform/apex was divided into vertical (depth deviation) and lateral deviations according to the longitudinal axis of the planned implant. Moreover, the lateral deviation was further divided into mesio-distal and bucco-lingual deviations by the previously created coordinate system of the corresponding implant site (Figure 5). A schematic diagram of the flow of experimental steps was presented in Figure 6.

### 2.4. Statistical Analysis

The number of 30 implants of each site group was calculated with a sample size calculation, using the effect size of 0.5, 80% power, and 5% α-error from a pilot study. Data analysis was made using STATA 14 statistics software (StataCorp, College Station, TX, USA). The mean value, standard deviation, and range were used to describe the quantitative data. The distributions of the deviations were presented with box plots. Intergroup differences were evaluated with the one-way analysis of variance (ANOVA) test, and Tukey’s method was used for multiple comparison analysis for the ANOVA results. A Bonferroni adjustment was used for multiple testing within one article, and *p* values smaller than 0.01 were deemed to be statistically significant.

## 3. Results

For the total number of implants, and each of the implant site groups, the deviations between the planned and placed implant position are presented and compared in Table 1; Table 2. No significant differences were found for any of the global, lateral, depth, and angular deviations among the three implant sites (Table 1). However, in the comparison of the mesio-distal and bucco-lingual deviations, significant differences were found in bucco-lingual deviations at the implant platform among the three implant sites (*p* < 0.0001) (Table 2). The bucco-lingual deviation at the implant platform of implants placed in the #47 position was significantly larger than the #46 and #36 position. The mean bucco-lingual deviation value of the #47 position (0.43 ± 0.19 mm) was twice as large as the #46 position (0.21 ± 0.14 mm), and almost twice as large as the #36 position (0.24 ± 0.25 mm).

**Figure 7:**
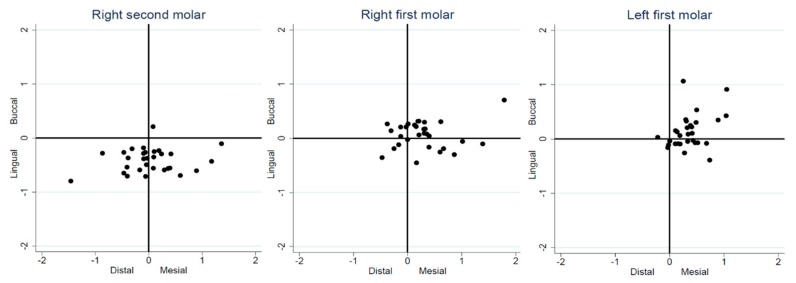
The distributions of the lateral deviations at the implant platform of every dentist in each implant site group.

The distributions of the lateral deviations at the implant platform of every dentist in each implant site group are presented in Figure 7. For the #47 site, all the bucco-lingual deviations at the implant platform were toward the lingual side, that is, the left side of the patient, except for one dentist. This lingually deviated tendency was also found at the implant apex. For the #46 site, no evident drift direction was found for the lateral deviations. However, for the #36 site, the lateral deviations tended to be more congregated than #46 and #47 sites, and most of the deviations of larger magnitude were towards the mesial or buccal direction at both the implant platform and apex.

**Figure 8:**
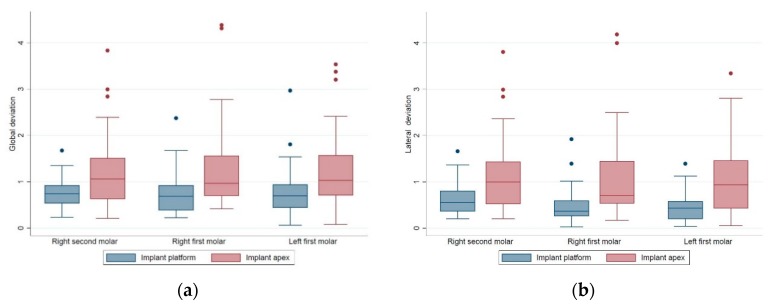
The box plots of the (**a**) global deviations and (**b**) lateral deviations at the implant platform and apex of each implant site group.

**Figure 9:**
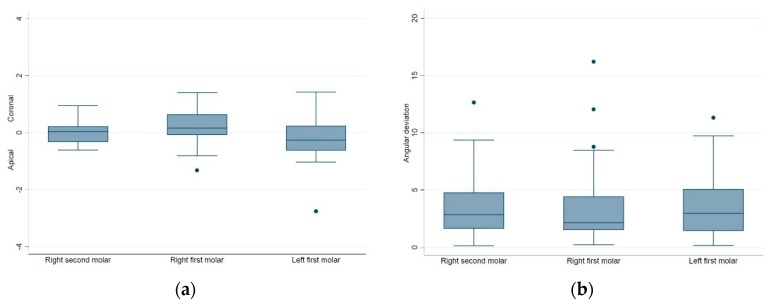
The box plots of the (**a**) depth deviations and (**b**) angular deviations of each implant site group.

For each of the implant site groups, the distributions of the global deviations at the implant platform/apex, the lateral deviations at the implant platform/apex, the depth deviations, and the angular deviations are presented respectively in the box plots in Figure 8; Figure 9. In general, the distributions of all the positional and angular deviations of each implant site group showed no remarkable difference from each other. For the depth deviations, implants placed in the #36 site tended to be deeper than the planned position (median value: −0.26 mm), and implants placed in the #46 site tended to be shallower than the planned position (median value: 0.16 mm). Implants placed in the #47 site tended to be equally distributed around the planned depth (median value 0.02 mm), with a more congregated distribution than the #36 and #46 site (Figure 9a).

## 4. Discussion

To the best of our knowledge, this was the first study to investigate the accuracy of sGIS using a combination tooth-and-bone supported SLA surgical guide in a distal extension situation. The results of this study revealed that, for mandibular partial edentulism of molars, placing dental implants in the first molar site by the combination tooth-and-bone supported surgical guide is as accurate as by a bilaterally tooth-supported surgical guide. The results support the null hypothesis (*p* > 0.01).

However, when placing the second distal implants via the same surgical guide, a significant larger bucco-lingual deviation at the implant platform was found when compared to the first distal implant. Moreover, almost all the bucco-lingual deviations of the second distal implant were toward the lingual (left) side when operated by the right-handed surgeons, suggesting a left-sided bending of the surgical guide during the simulation surgery. The depth deviations of the distal extension situation sites were not significantly different from the bilaterally tooth-supported site.

Most of the studies investigating the accuracy of sGIS measured the global or the lateral deviations between placed and planned implants, without further identifying the directional errors of the deviations [17]. In the present study, the measured lateral deviations were further divided into mesio-distal and bucco-lingual deviations according to each implant location. This helped to detect the slight differences which may not be distinguished in the earlier studies. In addition, in vitro simulation implant surgeries performing on dental models were used for better control of the study variables; hence, the possible confounding parameters of a clinical trial can be excluded. In this study, the mean global deviations of 90 implants at the implant platform/apex were 0.76/1.28 mm, and the mean angular deviation was 3.64°. In a systematic review and meta-analysis study including 24 clinical and preclinical studies, Tahmaseb et al. [2] reported a mean global error of 1.12/1.39 mm at the implant platform/apex and a mean angular error of 3.89°. Therefore, the results of this study were reasonable and coinciding with the literatures.

In the literature, the accuracy of sGIS employing the one-end supported surgical guide has previously been examined. Kim et al. [18] reported no significant differences in computer-based implant placement accuracy between distal extension and tooth-end cases in their model study. In their study, however, the implants were placed into the model using the flapless technique, with the surgical guide in contact with the silicone-based artificial gingival material, which may offer some support of the free-end guide and influence the results. Moreover, only the 3D-distance and vertical deviation were used to estimate the implant placement accuracy in their study, without further analysis of the lateral and specific directional (mesio-distal or bucco-lingual) errors.

In a clinical study, Behneke et al. [12] evaluated the accuracy of 132 implants in 52 partially edentulous patients using laboratory-based computer surgical guides. No significant effect on accuracy could be found for placing implants with free ending templates in the shortened dental arch group, compared with the interrupted dental arch group using the mesial and distal tooth-supported templates. In their study, nevertheless, half of the implants were installed using a flapless approach, and only 24 (18%) implants were installed with fully guided implant placement. Additionally, the number of missing teeth of the shortened dental arch and interrupted dental arch was not mentioned, making the precise comparisons of the accuracies among different implant sites difficult.

On the other hand, other studies investigating the accuracy of sGIS agreed that implants placed via free-ending templates often showed higher deviations [13,14,15,19]. In the free-end situation, micro-movements of the surgical guide may arise during the drilling process [20]. In addition, tilting and bending of the free-end surgical guide could occur during the drilling and implant placement, leading to a more significant inaccuracy. Behneke et al. [12] mentioned that such tilting could be prevented by using a rigid template material and the relining of the templates to obtain a sufficient stiffness of the guide. Nevertheless, to print the surgical guide with a more rigid material, such as metals, inevitably increases the cost. The surgical guide made of metals can be difficult to adjust if necessary. In addition, relining the guide could be impractical for some clinical situations, especially when an open flap surgery is desirable.

The authors advocated a design combined with tooth support and bone support. By adding a rigid supporting strut in the distal part, the one-end supported guide was converted to a combination tooth-and-bone supported guide with bilateral support. The results of the present study revealed that all the positional and angular deviations of the three implant sites, except the bucco-lingual deviations at the implant platform of the #47 position, were not significantly different from each other, including the depth deviations. This suggests that, by providing an anchor of surgical guide on the distal free-end side, this design is beneficial to the accuracy of the sGIS, possibly by reducing the tilting or the downward deformation of the surgical guide during drilling and implant placement.

In the present study, the bucco-lingual deviations at the implant platform of the #47 site were significantly larger than the #36 and #37 sites, whereas the bucco-lingual deviations at the implant apex and the angular deviations of the three implant sites were not significantly different from each other. Incorrect intraoral seating of the surgical guide could affect accuracy [1]. In a study of mucosa-supported surgical guides on edentulous jaws, Van de Wiele et al. [21] observed a smaller inter-implant deviation than the global deviation, clearly indicating the inaccuracy was mostly due to incorrect positioning of the guide. In this situation, the sequence of osteotomy or implant placement may affect the final accuracy of all implants. The present study used tooth-supported surgical guides that were not fixed by fixation screws. The fixture mount did not anchor the surgical guide after placing an implant either; hence, the effect of drilling or implant insertion sequence should be minimum.

Moreover, almost all the bucco-lingual deviations of the #47 site were towards the lingual side (the left side of the patient), but this phenomenon was not seen for the #36 and #46 sites. Considering all the dentists in this study used their right hand in performing the simulation surgery, this suggested a left-side bending tendency of the surgical guide while operated by right-handed surgeons. The magnitude of this deformation did not influence the bucco-lingual accuracy in the first implant site of the free-end; however, the second distal implant site was affected. Hence, the rigidity against bucco-lingual deformation of this combination tooth-and-bone supported guide should be improved. Increasing the buccolingual thickness of the surgical guide may be helpful, and further studies are needed to examine the effect.

The influence of the dominant hand of the surgeons on the accuracy of sGIS has been discussed in the literature. Van de Wiele et al. [21] found that right-handed surgeons placed implants significantly more accurately on the right side of the patients than on the left side. Van Assche et al. [3] suggested a deviation of the implants to the right or the mesial was associated with right-handed surgeons, especially for the more distal implants. However, the present study did not find the effects mentioned above.

With regard to the distribution of the lateral deviations, they were more congregated at the #36 site than the implant sites in distal extension situation. This reveals that for a single tooth gap, the nearby mesial and distal teeth provide more stability for the surgical guide from shifting or deformation. Moreover, large lateral deviations could still be found, especially in the #46 and #47 sites. All the large lateral deviations of the #46 and #47 sites were mesio-distal oriented. Because the distal supporting strut may improve the downward bending of the surgical guide, this could possibly be due to the limited angle of placement of the drill head in the posterior teeth area. Verhamme et al. [22] mentioned that a limited mouth opening might cause mesial or palatal leaning of the drill head.

The position and the angulation of the drill within the sleeve during the osteotomy procedure is essential for achieving accuracy of sGIS [23]. Accordingly, the mechanical tolerance of the drill and the sleeve of the surgical guide, the mouth-opening limitations of the patient, and the technical skills of the surgeon could significantly affect the accuracy of sGIS [3,24,25]. Repeated use of the drills and guide sleeves could increase the mechanical tolerance, and hence substantially decrease the accuracy of implant placements [26]. In this study, each dentist used a brand-new drill for the final drilling procedure to minimize the errors from using dull drills during the osteotomy procedure. Therefore, the influence of the mechanical tolerance of the surgical guide system should be minimal in this study.

Another possible explanation for the few large deviations is the technical skills of the surgeon. In an in vitro study, Laederach et al. [27] concluded that the clinician might have considerable influence on the accuracy of sGIS when applying eccentric forces. Considering the simulation was executed in a well-controlled environment, the few large deviations could be attributed to the personal technical skills of individual surgeon. Van Assche and Quirynen [23] mentioned that maintaining the drill in a parallel and central position is essential for achieving accuracy with sGIS. It is important to further investigate the particular reasons that influence the performance of individual surgeons.

In order to understand the realistic performance of the combination tooth-and-bone supported SLA surgical guide, we recruited 30 dentists instead of one or two experienced surgeons to conduct the study. In this study, we did not find any significant differences of accuracy between the experienced and inexperienced dentists. However, the mean values of deviations and the existence of few large deviations were coinciding with the results of several systematic reviews [2,3,4]. Furthermore, the sample size and 80% statistical power of this study were enough to detect the association in the deviations and their significant difference among implant sites by one-way ANOVA. Hence, the existence of few large deviations should be representative of the real-world performance, and do not affect the interpretations of the results of this study.

In this study, an in vitro model experiment was used for better control of the confounding parameters. We attempted to minimize the error by optically scanning every model to select the models with identical outlines to the master model used for making the surgical guides. Artificial gingiva material was also removed to eliminate the potential influence of the reflected flap. However, in real patients, the reflected soft tissue could affect the correct seating of the surgical guide and influence the accuracy. Furthermore, the surface texture of the model could be smoother than the cortical bone; hence, the surgical guide could be prone to shift in this study when eccentric forces are applied.

In addition, the distal bone contour of the edentulous ridge in real patients could also be irregular or ambiguous on the CT or CBCT image, making the correct design of the supporting strut difficult. Another limitation of this study was that the effect of the proposed combination tooth-and-bone supported stereolithographic surgical guide could not be verified by comparing the surgical guide with and without the supporting strut. Randomized clinical trials are needed in the future to validate the effect and accuracy of this design.

## 5. Conclusions

Within the limits of the present study, the following conclusions are drawn based on the results of the experimental work:

1. Placing dental implants in the first molar site via the combination tooth-and-bone supported surgical guide is as accurate as via a bilaterally tooth-supported surgical guide.
2. Instead of apical tilting, bucco-lingual bending of the combination tooth-and-bone supported surgical guide could occur, consequently causing a more significant bucco-lingual deviation when placing the second distal implant via the surgical guide.
3. In distal extension situation of missing mandibular molars, adding a bone-supported strut in the distal part of the surgical guide can be beneficial to the accuracy of the sGIS, possibly by reducing the tilting or the downward deformation of the surgical guide.

## Figures and Tables

**Table 1:** Global, lateral, depth, and angular deviations between the planned and placed implant position.

Implant Site Group	Implant Number	Deviations at Implant Platform		Deviations at Implant Apex		Depth Deviation (mm)	Angular Deviation (degree)
		Global (mm)	Lateral (mm)	Global (mm)	Lateral (mm)		
Total	90	0.76 ± 0.46	0.53 ± 0.36	1.28 ± 0.94	1.13 ± 0.89	0.45 ± 0.42	3.64 ± 3.11
(range)		(0.06–2.97)	(0.02–1.92)	(0.07–4.38)	(0.05–4.19)	(0.01–2.76)	(0.12–16.21)
#36	30	0.78 ± 0.57	0.47 ± 0.33	1.29 ± 0.88	1.10 ± 0.80	0.57 ± 0.53	3.67 ± 2.73
(range)		(0.06–2.97)	(0.04–1.39)	(0.07–3.53)	(0.05–3.35)	(0.05–2.76)	(0.16–11.32)
#46	30	0.75 ± 0.47	0.49 ± 0.39	1.30 ± 1.00	1.12 ± 1.01	0.49 ± 0.39	3.68 ± 3.66
(range)		(0.22–2.37)	(0.02–1.92)	(0.42–4.38)	(0.17–4.19)	(0.03–1.39)	(0.23–16.21)
#47	30	0.75 ± 0.33	0.63 ± 0.35	1.24 ± 0.85	1.15 ± 0.88	0.31 ± 0.24	3.55 ± 2.97
(range)		(0.23–1.67)	(0.21–1.66)	(0.21–3.83)	(0.20–3.81)	(0.01–0.94)	(0.12–12.65)
*p* Value		0.9506	0.1850	0.9619	0.9728	0.0407	0.9835

#36: Mandibular left first molar; #46: Mandibular right first molar; and #47: Mandibular right second molar.

**Table 2:** Mesio-distal and bucco-lingual deviations between the planned and placed implant position.

Implant Site Group	Implant Number	Deviations at Implant Platform		Deviations at Implant Apex	
		Mesio-Distal (mm)	Bucco-Lingual (mm)	Mesio-Distal (mm)	Bucco-Lingual (mm)
Total	90	0.39 ± 0.36	0.29 ± 0.22	0.94 ± 0.88	0.47 ± 0.42
(range)		(0.00–1.79)	(0.02–1.06)	(0.00–4.18)	(0.02–2.28)
#36	30	0.38 ± 0.28	0.24 ± 0.25 ^b^	0.89 ± 0.67	0.54 ± 0.58
(range)		(0.01–1.05)	(0.03–1.06)	(0.01–2.60)	(0.02–2.28)
#46	30	0.41 ± 0.40	0.21 ± 0.14 ^b^	0.99 ± 1.03	0.40 ± 0.28
(range)		(0.00–1.79)	(0.02–0.71)	(0.00–4.18)	(0.04–1.18)
#47	30	0.39 ± 0.39	0.43 ± 0.19 ^a^	0.94 ± 0.94	0.49 ± 0.35
(range)		(0.03–1.46)	(0.11–0.80)	(0.02–3.62)	(0.02–1.18)
*p* Value		0.9463	<0.0001 *	0.9061	0.4707

* *p* < 0.01, ANOVA test, **^a b^** significant difference by Tukey multiple comparisons. #36: Mandibular left first molar; #46: Mandibular right first molar; and #47: Mandibular right second molar.
